# The Transcribed-Ultraconserved Regions: A Novel Class of Long Noncoding RNAs Involved in Cancer Susceptibility

**DOI:** 10.1100/tsw.2011.35

**Published:** 2011-02-03

**Authors:** Paola Scaruffi

**Affiliations:** Center of Physiopathology of Human Reproduction, Department of Obstetrics and Gynecology, “San Martino” Hospital, Genoa, Italy

**Keywords:** ultraconserved regions, noncoding RNA, gene expression

## Abstract

During recent years, novel approaches and new technologies have revealed a startling level of complexity of higher eukaryotes' transcriptome. A large proportion of the transcriptional output is represented by protein noncoding RNAs (ncRNAs) that arise from the “dark matter” of the genome. Focus on such sequences has revealed numerous RNA subtypes with several functions in RNA processing and gene expression regulation, and deep sequencing studies imply that many remain to be discovered. This review gives a picture of the state of the art of a novel class of long ncRNA known as transcribed-ultraconserved regions (T-UCRs). Most recent studies show that they are significantly altered in adult chronic lymphocytic leukemias, carcinomas, and pediatric neuroblastomas, leading to the hypothesis that UCRs may play a role in tumorigenesis and promising innovative future T-UCR—based therapeutic approaches.

## INTRODUCTION

Since their introduction in the mid-1990s, microarrays have rapidly become a high-throughput method of gene expression analysis in relation to physiology, development, and disease. Moreover, together with sequencing of the human genome as well as those of model organisms, they largely contributed to the exploration of the complexities of eukaryotic genomes[1]. In the last few years, there has been increasing evidence that ~98% of human DNA is transcribed into molecules that are protein noncoding RNAs (ncRNAs)[2,3]. Such a startling finding has revolutionized the central dogma of molecular biology, according to information flows from DNA to protein through RNA as its intermediary[4]. From there, it was easy to generalize that "one gene equals one protein, one function". Generally, this holds true in prokaryotes, whose genomes consist of tightly packed protein-coding sequences, whereas complex eukaryotes have absolutely different patterns of functional regulation. Thus, the modern view of the eukaryotic RNA world involves many ncRNAs, which process and regulate other RNA molecules by cleavage, nucleotide modification, transcription, and degradation[5]. Numerous subtypes of ncRNAs participate in such RNA settings, including rRNAs, mRNAs, tRNAs, mitochondrial ncRNAs[6], small nuclear RNAs (snRNAs), small nucleolar RNAs (snoRNAs), several classes of regulatory RNAs involved in RNA interference (RNAi), and many long intergenic ncRNAs (lincRNAs)[7,8,9], such as *HOTAIR*[10]. In the last years, significant progress has been made on functional annotation of a particular class of small ncRNAs, namely microRNAs (miRNAs). These are short RNA molecules, on average only 22 nucleotides long, that both work as negative post-transcriptional regulators of gene expression through imperfect binding to target mRNAs[11] and also induce up-regulation of target mRNAs, i.e., during the cell cycle[12]. Conversely, the functionality of long ncRNAs is still elusive. Some long ncRNAs are located and transcribed within the intergenic stretches and they associate with chromatin-modifying complexes to alter gene expression[13,14]. The majority of long ncRNAs are transcribed as complex networks of overlapping sense and antisense transcripts that often include protein-coding genes[15]. Genomic sequences of these transcriptional foci are often shared within a number of different coding and noncoding transcripts in the sense and antisense directions, giving rise to a complex hierarchy of overlapping isoforms[16].

Many small RNAs, such as miRNAs or snoRNAs, and a representative set of human long ncRNAs, exhibit strong conservation at the levels of sequence, promoter, and splicing across diverse species[17,18], which is indicative of positive selection[19].

## THE ULTRACONSERVED REGIONS

Ultraconserved regions (UCRs) were discovered in 2004 by bioinformatics comparisons of the mouse, rat, and human genomes[20]. They are 481 genomic elements longer than 200 bp (range: 200–779 bp) that are absolutely conserved (100% identity with no insertions or deletions) among the three species. Even single-nucleotide polymorphisms (SNPs) are under-represented within UCRs[20,21] and ultraconserved elements do not accumulate mutations in somatic cells in conditions of genomic instability[22]. Such extreme conservation could be a sign of a strong purifying selection[23]. As a proof of principle of the high levels of conservation of UCRs during evolution, a distal enhancer and an ultraconserved exon, originating from the short interspersed repetitive element retroposon family active in lobe-finned fishes and terrestrial vertebrates more than 400 million years ago, have been found still active in a ‘‘living fossil” Indonesian coelacanth[24].

Originally, focusing on overlap with protein-coding regions based on hg17 genome assembly, 111 (23%) UCRs were categorized as exonic and 256 (53%) as nonexonic, whereas the remaining 114 (24%) UCRs, for which the evidence for transcription was inconclusive, were called possibly exonic[20]. Recently, Mestdagh and colleagues[25] reannotated all UCR sequences using the more recent genome build hg18 and reorganized them into five different categories (intergenic, intronic, exonic, partly exonic, exon containing) by matching their location to that of the human RefSeq genes. The new genomic categories are unambiguously defined and provide a more detailed genomic annotation for each UCR: 38.7% UCRs, located between genes, are termed as intergenic; 42.6% as intronic; 4.2% as exonic; 5% as partly exonic; and 5.6% as exon containing. For a few UCRs (3.9%), the genomic annotation varies because of host gene splice variants and these UCRs are categorized as “multiple”.

Intergenic UCRs are often found in “gene deserts” that extend more than 1 Mb. Precisely, 140 nonexonic elements are more than 10 kb away from any known gene, and 88 are more than 100 kb away[20]. The set of 156 annotated genes that flank intergenic UCRs is significantly enriched for genes involved in early developmental tasks, suggesting that many of the associated UCRs may be distal enhancers of these developmental genes[26]. For instance, one of these elements (uc.351) is contained in an enhancer situated about 225 kb upstream of *DACH* (homolog of the *Drosophila* dachshund gene), known to be involved in the development of brain, limbs, and sensory organs[20,27]. Also, intronic UCRs are often associated with developmental genes. These include the neuroretina-specific enhancer in the fourth intron of *PAX6* (uc.328)[20,28].

There are 93 known protein-coding genes that overlap with exonic UCRs. Such genes show significant functional enrichment for RNA binding (i.e., *HNRPK*, *HNRPH1*, *HNRPU*, *HNRPDL*, *HNRPM*, *SFRS1*, *SFRS3*, *SFRS6*, *SFRS7*, *SFRS10*, *SFRS11*, *TRA2A*, *PCBP2*, and *PTBP2*), regulation of splicing, RNA recognition motifs (including the six members of the *SFRS* family), as well as DNA binding motifs, in particular the Homeobox domain. These attributes are enriched in the 225 protein-coding genes that are near the nonexonic UCRs as well, although less significantly, suggesting that exonic UCRs may be specifically associated with RNA processing and nonexonic elements with regulation of transcription at DNA level[20].

The UCRs are frequently located at fragile sites and cancer-associated genomic regions (CAGRs), such as minimal regions of amplification and of loss of heterozygosity[29] (Supplementary Table 1). Intriguingly, those UCRs differentially expressed in human cancers are located in CAGRs specifically associated with that type of cancer. This is the case of uc.349A and uc.352 differentially expressed between normal and leukemic CD5-positive cells[30]: they are located within the 13q21.33–q22.2 chromosomal region, which has been linked to susceptibility to familial chronic lymphocytic leukemia[31]. Also, in pediatric neuroblastoma (NB) tumors, expression of seven UCRs correlated to their copy-number status[25]. Together, these data suggest that not only the protein-coding genes, but also the UCRs located in the CAGRs, could be candidate players for cancer susceptibility.

## TRANSCRIBED-UCRs

A large fraction of UCRs are transcribed (so-called transcribed-UCRs, T-UCRs) in normal human tissues, and their expression levels show both a ubiquitous (for 34% of T-UCRs) and a tissue-specific pattern[30]. The T-UCRs show predominant transcription from one strand and only 9% of them are bidirectionally transcribed[30].

Interestingly, in addition to microarrays, Northern blot, and reverse transcription-quantitative real-time PCR (RT-qPCR)[29], a linear isothermal Ribo-SPIA™ RNA amplification method enables sensitive and accurate high-throughput interrogation of all 481 T-UCRs[32]. This is particularly important since transcription regulation studies are increasingly conducted in small samples of potential clinical interest, such as tumor biopsies, laser capture microdissected or sorted cell populations, when limited starting RNA amounts are available.

## T-UCR AND TUMORS

Calin et al.[30] were the first to investigate the expression of UCRs in human cancers, focusing on chronic lymphocytic leukemia, the most frequent adult leukemia in the Western world[33], on colorectal carcinoma, one of the most common cancers in industrialized countries[34], and on hepatocellular carcinoma, the most rapidly increasing type of cancer in the U.S.[35]. They found that, for all the tumor types examined, the malignant cells have a unique spectrum of expressed UCRs when compared with the corresponding normal cells, suggesting that variations in T-UCR expression are involved in the malignant process. Moreover, distinct T-UCR expression signatures were differentially expressed in leukemias and carcinomas, and thus they might offer a novel strategy for cancer diagnosis and prognosis.

Recently, we investigated T-UCR expression in NB tumors[36]. The NB is a pediatric tumor of the sympathetic nervous system characterized by a remarkable heterogeneous clinical behavior[37]. Patients with localized NB have a favorable outcome and in infants with disseminated stage-4 tumor, the progression of disease is generally halted by good response to therapy. Conversely, only 20–30% of children older than 12–18 months of age with a stage-4 tumor show progression free– and overall survival longer than 60 months, despite multimodal therapeutic protocols[38]. In recent years, several prognostic signatures derived from gene expression profiles, DNA abnormalities, and miRNAs have been proposed as sensitive indicators of tumor progression in NB patients[39,40,41,42,43]. Although efforts have been performed in order to validate each gene classifier on independent patient cohorts[44,45], the major challenges remain to identify additional tumor-specific prognostic markers for improved risk estimation at the time of diagnosis, especially in high-risk NB patients. For the first time, we defined a signature based on 28 T-UCRs that is associated with good outcome in noninfant patients diagnosed with metastatic NB[36]. More recently, Mestdagh et al.[25] confirmed that T-UCRs are widely expressed in NB tumors and correlate to clinical-genetic parameters, such as *MYCN* oncogene status.

As regarding dysregulation of T-UCR transcription in cancer, Calin et al[30] demonstrated that transcription of tumor-associated T-UCRs in leukemias is negatively regulated by direct interaction with miRNAs. Similarly, we found negative correlations between expression values of nine specific T-UCRs and five predicted interactor miRNAs of the signature able to differentiate between long- and short-surviving high-risk NB patients[36]. In both studies, sequence complementarity gives rise to several miRNA:UCR interacting pairs, indicating complex redundancy in regulatory mechanisms between miRNAs and T-UCRs. Accordingly, these findings provide support for a model in which both coding and noncoding genes are involved in and cooperate in human tumorigenesis. Notably, it is now easily possible to match miRNA and UCR sequences by a specific database, namely Ucbase & miRfunc, which provides UCR sequence data and shows miRNA function[46].

To gain further insight into the initiation and regulation of T-UCR transcription, Mestdagh et al.[25] evaluated the chromatin state of the T-UCR genomic neighborhood. Both inter- and intragenic T-UCRs are significantly associated with active trimethylation of lysine 4 of histone H3 (H3K4me3), a marker for transcriptional initiation[47,48], but with a different distribution as compared with protein-coding genes, suggesting a difference in transcriptional organization between T-UCRs and protein-coding genes. In addition, H3K4me3 distance distributions for miRNAs and T-UCRs appear similar, suggesting common features of transcription organization for these two classes of ncRNA, with initiation sites several kilobases away[49].

Finally, epigenetic mechanisms as potential regulators of T-UCR expression have been evaluated. We found that 78% of intragenic T-UCRs deregulated in high-risk NBs are associated with CpG islands in the promoter region of their own host genes[36]. Therefore, much like CpG island hypermethylation-mediated silencing of miRNAs with tumor-suppressor features contributes to human cancer[50], the global DNA hypermethylation events in unfavorable NB[51,52] may also affect T-UCR-host genes, and thus silence T-UCRs with a potential oncogenic role. Recently, a pharmacological and genomic approach confirmed the possible existence of an aberrant epigenetic pattern of T-UCRs. Indeed, Lujambio and coauthors[53] observed that while almost half of the UCR-associated CpG islands are unmethylated in all tissues, the other half show tissue-specific UCR CpG island methylation, as occurs with promoter CpG islands of coding genes[54] and miRNAs[55]. Moreover, treatment of cancer cells with the DNA methylation inhibitor 5-aza-2-deoxycytidine disclosed that epigenetic inactivation by CpG island hypermethylation of a subset of T-UCRs occurs in a wide spectrum of human cancer cell lines and primary tumors. Taken together, these findings support a model in which epigenetic disruption of T-UCRs constitutes a hallmark of human tumorigenesis. Accordingly, tumor-specific CpG island hypermethylated UCRs may be useful biomarkers of disease.

Table 1 summarizes the main studies on the field of UCRs and cancer, and the main findings of each of these studies.

## FUNCTION OF THE ULTRACONSERVED ELEMENTS

The remarkably high degree of conservation across species implies that UCRs may have a fundamental functional importance for the ontogeny and phylogeny of mammals and other vertebrates. Although UCRs are significantly depleted among segmental duplications and copy-number variants[56], deletion of some of these regions in knock-out mice was not associated to any notable phenotype abnormality[57]. Therefore, the role of UCRs in viability is still controversial.

**TABLE 1**
**List of Studies on the Field of UCRs and Cancer and Main Findings of Each**

| Ref. | Tumor Type | Main Findings |
|---|---|---|
| Calin et al.[30] | Chronic lymphocytic leukemia<br>Colorectal carcinoma<br>Hepatocellular carcinoma | Identification of distinct tumor-associated UCR expression signatures.<br>UCRs are frequently located at fragile sites and genomic regions involved in cancers.<br>Expression of certain UCRs may be regulated by miRNAs abnormally expressed in human chronic lymphocytic leukemia.<br>Inhibition of overexpressed uc.73A induces apoptosis in colon cancer cells. |
| Scaruffi et al.[36] | Neuroblastoma | Identification of a signature based on T-UCR expression that is associated with good outcome in noninfant patients diagnosed with metastatic neuroblastoma. |
| Mestdagh et al.[25] | Neuroblastoma | Correlations between specific T-UCR expression levels and clinicogenetic parameters.<br>Assignments of T-UCRs to cellular processes such as *TP53* response, differentiation and proliferation. |
| Lujambio et al.[53] | Colon cancer<br>Breast cancer<br>Lung cancer<br>Melanoma<br>Leukemia<br>Lymphoma | Hypermethylation of T-UCR CpG islands. |

Based on the evidence of most recent studies, UCRs are believed to be important in vertebrate genomes, i.e., for long-range enhancer-like activity[58,59,60] (Supplementary Table 2), for homeostatic maintenance of splicing factor expression levels involved in post-transcriptionally gene regulation by alternative splicing coupled with nonsense-mediated mRNA decay[61,62], for regulation of transcription, both as epigenetic modification marks[63,64], and as transcriptional coactivators[65].

In cancer cells, T-UCRs might act as oncogenes. Indeed, functional analysis involving small interfering RNAs have identified one T-UCR in colorectal cancer, namely uc.73A, to be oncogenic by increasing the number of malignant cells as a consequence of reduced apoptosis[30]. To go into more depth of the processes in which T-UCRs are involved, Mestdagh and colleagues[25] implemented an integrative genomic workflow to infer putative T-UCR functions using Gene Set Enrichment Analysis[66] and validated them using *in vitro* systems. For a large number of T-UCRs, they observed widespread association to numerous cancer-related cellular functions and pathways, such as proliferation, apoptosis, and differentiation. For example, the most prominent cluster identified using this methodology contained several T-UCRs significantly related to the expression of protein-coding genes involved in cell cycle, DNA replication, and DNA repair.

## CONCLUSION AND FUTURE PERSPECTIVES

It has been well accepted that T-UCRs are regulatory elements within the RNA-processing machinery that also play a critical role in human diseases such as cancer. Indeed, malignant cells show specific alterations not only at genes coding for oncoproteins or tumor suppressors, but also at several classes of ncRNAs. Dysregulation of T-UCRs is a common feature of human cancer. It offers the prospect of defining both tumor-specific signatures of T-UCRs and tumor-specific methylated UCRs that are associated with diagnosis, prognosis, and response to treatments. Above all, aberrant UCR methylation in the transformed cells might provide a molecular basis for the innovative therapeutic use of DNA-demethylating compounds in the treatment of cancer patients. As a proof of principle, restoration of expression of a down-regulated T-UCR, or, alternatively, inhibition of an overexpressed T-UCR by a small interfering RNA approach could reverse the tumor phenotype. Moreover, localization of such ncRNA within CAGRs could open the way for starting T-UCR–based therapy trials.

## ACKNOWLEDGMENTS

This work was supported by Fondazione Italiana per la Lotta al Neuroblastoma and Ministero dell'Università, Ricerca Scientifica e Tecnologica.

**SUPPLEMENTARY TABLE 1**
**Annotation of T-UCR Located at CAGR According to Genome Build hg18**

| UCR Name | Location | Start (bp) | End (bp) |
|---|---|---|---|
| uc.1 | Chromosome 1 | 10520284 | 10520490 |
| uc.2 | Chromosome 1 | 10655130 | 10655336 |
| uc.3 | Chromosome 1 | 10673752 | 10673976 |
| uc.4 | Chromosome 1 | 10680836 | 10681194 |
| uc.5 | Chromosome 1 | 10703938 | 10704151 |
| uc.6 | Chromosome 1 | 10717708 | 10718008 |
| uc.7 | Chromosome 1 | 10758720 | 10758975 |
| uc.8 | Chromosome 1 | 10774405 | 10774620 |
| uc.9 | Chromosome 1 | 10847997 | 10848198 |
| uc.10 | Chromosome 1 | 10888161 | 10888435 |
| uc.21 | Chromosome 1 | 48885573 | 48885807 |
| uc.22 | Chromosome 1 | 50778819 | 50779126 |
| uc.23 | Chromosome 1 | 50808365 | 50808593 |
| uc.24 | Chromosome 1 | 50871733 | 50872068 |
| uc.25 | Chromosome 1 | 50938622 | 50938856 |
| uc.26 | Chromosome 1 | 63142234 | 63142445 |
| uc.27 | Chromosome 1 | 63142468 | 63142757 |
| uc.28 | Chromosome 1 | 70469301 | 70469655 |
| uc.35 | Chromosome 1 | 97774870 | 97775074 |
| uc.38 | Chromosome 1 | 162206579 | 162206802 |
| uc.39 | Chromosome 1 | 162290159 | 162290514 |
| uc.40 | Chromosome 1 | 162904586 | 162904832 |
| uc.62 | Chromosome 2 | 60634186 | 60634419 |
| uc.63 | Chromosome 2 | 61606005 | 61606282 |
| uc.66 | Chromosome 2 | 73028511 | 73028757 |
| uc.95 | Chromosome 2 | 171279776 | 171280026 |
| uc.96 | Chromosome 2 | 172528920 | 172529180 |
| uc.97 | Chromosome 2 | 172530877 | 172531318 |
| uc.98 | Chromosome 2 | 172664764 | 172665001 |
| uc.99 | Chromosome 2 | 172666627 | 172667024 |
| uc.108 | Chromosome 2 | 176648603 | 176648976 |
| uc.109 | Chromosome 2 | 177211584 | 177211807 |
| uc.110 | Chromosome 2 | 236736121 | 236736363 |
| uc.111 | Chromosome 3 | 9446461 | 9446756 |
| uc.116 | Chromosome 3 | 70649114 | 70649319 |
| uc.117 | Chromosome 3 | 70954530 | 70954780 |
| uc.118 | Chromosome 3 | 70954782 | 70955000 |
| uc.135 | Chromosome 3 | 170316977 | 170317177 |
| uc.137 | Chromosome 3 | 181919551 | 181919935 |
| uc.139 | Chromosome 4 | 4574341 | 4574678 |
| uc.140 | Chromosome 4 | 12618968 | 12619190 |
| uc.141 | Chromosome 4 | 24138260 | 24138554 |
| uc.145 | Chromosome 4 | 105565762 | 105566009 |
| uc.146 | Chromosome 4 | 112135925 | 112136138 |

| | | | |
|---|---|---|---|
| uc.150 | Chromosome 5 | 3565621 | 3565882 |
| uc.151 | Chromosome 5 | 32415894 | 32416107 |
| uc.152 | Chromosome 5 | 50371395 | 50371595 |
| uc.173 | Chromosome 5 | 133754059 | 133754334 |
| uc.177 | Chromosome 5 | 170350234 | 170350490 |
| uc.178 | Chromosome 5 | 170350603 | 170350851 |
| uc.179 | Chromosome 5 | 170560817 | 170561035 |
| uc.180 | Chromosome 5 | 170561094 | 170561318 |
| uc.181 | Chromosome 5 | 170562084 | 170562361 |
| uc.182 | Chromosome 5 | 170635684 | 170635922 |
| uc.183 | Chromosome 5 | 171317125 | 171317360 |
| uc.193 | Chromosome 6 | 86378405 | 86378723 |
| uc.194 | Chromosome 6 | 94025785 | 94025985 |
| uc.203 | Chromosome 6 | 163911694 | 163911896 |
| uc.210 | Chromosome 7 | 26663603 | 26663859 |
| uc.211 | Chromosome 7 | 26695997 | 26696287 |
| uc.212 | Chromosome 7 | 27108463 | 27108667 |
| uc.213 | Chromosome 7 | 27149657 | 27149857 |
| uc.220 | Chromosome 7 | 96471852 | 96472108 |
| uc.221 | Chromosome 7 | 96479237 | 96479585 |
| uc.222 | Chromosome 7 | 113844407 | 113844607 |
| uc.223 | Chromosome 7 | 113845421 | 113845688 |
| uc.224 | Chromosome 7 | 113850255 | 113850549 |
| uc.225 | Chromosome 7 | 113860091 | 113860291 |
| uc.226 | Chromosome 7 | 113996554 | 113996758 |
| uc.227 | Chromosome 7 | 114082552 | 114082782 |
| uc.228 | Chromosome 7 | 114903933 | 114904197 |
| uc.229 | Chromosome 7 | 114921881 | 114922176 |
| uc.230 | Chromosome 7 | 115106697 | 115106934 |
| uc.231 | Chromosome 7 | 115369353 | 115369576 |
| uc.235 | Chromosome 8 | 25831838 | 25832064 |
| uc.243 | Chromosome 8 | 77853517 | 77853732 |
| uc.246 | Chromosome 8 | 119192399 | 119192682 |
| uc.265 | Chromosome 9 | 107158292 | 107158508 |
| uc.266 | Chromosome 9 | 108418113 | 108418355 |
| uc.267 | Chromosome 9 | 124093711 | 124093913 |
| uc.268 | Chromosome 9 | 124646649 | 124646899 |
| uc.269 | Chromosome 9 | 125577758 | 125577974 |
| uc.270 | Chromosome 9 | 127343890 | 127344167 |
| uc.271 | Chromosome 9 | 127344173 | 127344383 |
| uc.272 | Chromosome 9 | 127472409 | 127472621 |
| uc.273 | Chromosome 9 | 127557419 | 127557739 |
| uc.274 | Chromosome 9 | 127561692 | 127562018 |
| uc.275 | Chromosome 9 | 127623937 | 127624191 |
| uc.276 | Chromosome 9 | 127645633 | 127646064 |
| uc.277 | Chromosome 9 | 127647531 | 127647806 |
| uc.278 | Chromosome 9 | 127685986 | 127686222 |
| uc.279 | Chromosome 9 | 127712380 | 127712715 |

| | | | |
|---|---|---|---|
| uc.280 | Chromosome 9 | 127717827 | 127718046 |
| uc.282 | Chromosome 9 | 139162311 | 139162517 |
| uc.293 | Chromosome 10 | 102362659 | 102362901 |
| uc.294 | Chromosome 10 | 102363607 | 102364050 |
| uc.295 | Chromosome 10 | 102365090 | 102365298 |
| uc.296 | Chromosome 10 | 102405096 | 102405556 |
| uc.297 | Chromosome 10 | 102409210 | 102409573 |
| uc.298 | Chromosome 10 | 102437648 | 102438006 |
| uc.299 | Chromosome 10 | 102499425 | 102499634 |
| uc.300 | Chromosome 10 | 102537108 | 102537315 |
| uc.301 | Chromosome 10 | 102557781 | 102558064 |
| uc.302 | Chromosome 10 | 102969170 | 102969510 |
| uc.303 | Chromosome 10 | 103042417 | 103042688 |
| uc.304 | Chromosome 10 | 103072494 | 103072765 |
| uc.305 | Chromosome 10 | 103201425 | 103201729 |
| uc.306 | Chromosome 10 | 103202029 | 103202252 |
| uc.307 | Chromosome 10 | 103233973 | 103234204 |
| uc.308 | Chromosome 10 | 103235802 | 103236078 |
| uc.309 | Chromosome 10 | 103257021 | 103257288 |
| uc.310 | Chromosome 10 | 114394213 | 114394441 |
| uc.311 | Chromosome 10 | 120064392 | 120064610 |
| uc.312 | Chromosome 10 | 120066527 | 120066848 |
| uc.317 | Chromosome 10 | 131336328 | 131336545 |
| uc.318 | Chromosome 10 | 131581576 | 131581896 |
| uc.330 | Chromosome 11 | 66150472 | 66150678 |
| uc.333 | Chromosome 11 | 124149857 | 124150126 |
| uc.338 | Chromosome 12 | 52144756 | 52144978 |
| uc.339 | Chromosome 12 | 52357363 | 52357614 |
| uc.340 | Chromosome 12 | 52377099 | 52377357 |
| uc.341 | Chromosome 12 | 52669185 | 52669498 |
| uc.342 | Chromosome 12 | 52696761 | 52696987 |
| uc.343 | Chromosome 12 | 52708708 | 52709095 |
| uc.344 | Chromosome 12 | 52713153 | 52713406 |
| uc.345 | Chromosome 12 | 52733867 | 52734167 |
| uc.347 | Chromosome 13 | 70691989 | 70692197 |
| uc.348 | Chromosome 13 | 70961358 | 70961597 |
| uc.349 | Chromosome 13 | 71019303 | 71019505 |
| uc.350 | Chromosome 13 | 71154101 | 71154340 |
| uc.351 | Chromosome 13 | 71566901 | 71567155 |
| uc.352 | Chromosome 13 | 71592166 | 71592365 |
| uc.353 | Chromosome 13 | 71669554 | 71669876 |
| uc.355 | Chromosome 13 | 94416883 | 94417110 |
| uc.356 | Chromosome 13 | 96806821 | 96807071 |
| uc.357 | Chromosome 13 | 111764338 | 111764579 |
| uc.361 | Chromosome 14 | 28302886 | 28303152 |
| uc.362 | Chromosome 14 | 28418503 | 28418741 |
| uc.363 | Chromosome 14 | 28931070 | 28931334 |
| uc.364 | Chromosome 14 | 29782510 | 29782716 |

| | | | |
|---|---|---|---|
| uc.365 | Chromosome 14 | 29812113 | 29812390 |
| uc.366 | Chromosome 14 | 30452458 | 30452659 |
| uc.367 | Chromosome 14 | 32914214 | 32914511 |
| uc.368 | Chromosome 14 | 33138327 | 33138554 |
| uc.369 | Chromosome 14 | 33192368 | 33192580 |
| uc.370 | Chromosome 14 | 33272322 | 33272714 |
| uc.371 | Chromosome 14 | 35089940 | 35090235 |
| uc.372 | Chromosome 14 | 35112786 | 35113062 |
| uc.373 | Chromosome 14 | 35651558 | 35651951 |
| uc.374 | Chromosome 14 | 36785664 | 36785887 |
| uc.375 | Chromosome 14 | 36846971 | 36847270 |
| uc.379 | Chromosome 14 | 96501121 | 96501372 |
| uc.380 | Chromosome 14 | 96832347 | 96832578 |
| uc.381 | Chromosome 14 | 96949043 | 96949280 |
| uc.382 | Chromosome 15 | 33706204 | 33706403 |
| uc.383 | Chromosome 15 | 34607219 | 34607487 |
| uc.384 | Chromosome 15 | 34752685 | 34752950 |
| uc.385 | Chromosome 15 | 34972962 | 34973170 |
| uc.386 | Chromosome 15 | 35309301 | 35309503 |
| uc.413 | Chromosome 17 | 34820045 | 34820316 |
| uc.415 | Chromosome 17 | 44018905 | 44019111 |
| uc.416 | Chromosome 17 | 44025886 | 44026171 |
| uc.417 | Chromosome 17 | 44037311 | 44037532 |
| uc.418 | Chromosome 17 | 53437227 | 53437443 |
| uc.419 | Chromosome 17 | 53437714 | 53438002 |
| uc.444 | Chromosome 19 | 35186619 | 35187006 |
| uc.445 | Chromosome 19 | 35258275 | 35258584 |
| uc.446 | Chromosome 19 | 35439694 | 35439965 |
| uc.447 | Chromosome 19 | 35459621 | 35459893 |
| uc.458 | Chromosome 22 | 34478438 | 34478641 |

## SUPPLEMENTARY TABLE 2
## List of T-UCR with a Known Enhancer Activity

| | | | | | | | | | |
|---|---|---|---|---|---|---|---|---|---|
| uc.2 | uc.41 | uc.94 | uc.137 | uc.180 | uc.222 | uc.269 | uc.355 | uc.421 | uc.463 |
| uc.5 | uc.47 | uc.98 | uc.140 | uc.181 | uc.223 | uc.288 | uc.358 | uc.425 | uc.467 |
| uc.8 | uc.54 | uc.100 | uc.145 | uc.190 | uc.227 | uc.300 | uc.363 | uc.430 | uc.470 |
| uc.10 | uc.55 | uc.104 | uc.150 | uc.192 | uc.236 | uc.309 | uc.365 | uc.435 | uc.476 |
| uc.15 | uc.60 | uc.105 | uc.152 | uc.196 | uc.245 | uc.314 | uc.371 | uc.437 | uc.482 |
| uc.18 | uc.65 | uc.108 | uc.157 | uc.198 | uc.248 | uc.315 | uc.383 | uc.438 | |
| uc.19 | uc.67 | uc.109 | uc.163 | uc.204 | uc.249 | uc.316 | uc.388 | uc.444 | |
| uc.23 | uc.78 | uc.110 | uc.164 | uc.206 | uc.250 | uc.322 | uc.389 | uc.445 | |
| uc.25 | uc.82 | uc.112 | uc.165 | uc.207 | uc.252 | uc.325 | uc.392 | uc.447 | |
| uc.26 | uc.85 | uc.119 | uc.168 | uc.211 | uc.254 | uc.329 | uc.396 | uc.448 | |
| uc.27 | uc.87 | uc.123 | uc.170 | uc.214 | uc.260 | uc.334 | uc.400 | uc.449 | |
| uc.29 | uc.88 | uc.124 | uc.175 | uc.215 | uc.261 | uc.335 | uc.410 | uc.450 | |
| uc.31 | uc.92 | uc.125 | uc.179 | uc.220 | uc.262 | uc.353 | uc.411 | uc.462 | |
